# How do health content creators perform well? An integration research of short video and livestream behaviors

**DOI:** 10.3389/fpubh.2024.1446247

**Published:** 2024-10-02

**Authors:** Jing Liu, Qing Ye, Hong Wu, Rongyang Ma, Shanshan Guo, Han Long

**Affiliations:** ^1^Administrative Office, Yuebei People's Hospital, Medical Colledge, Shantou University, Shantou, China; ^2^Tongji Hospital, Tongji Medical College, Huazhong University of Science and Technology, Wuhan, China; ^3^School of Medicine and Health Management, Tongji Medical College, Huazhong University of Science and Technology, Wuhan, China; ^4^Tsinghua International Graduate School, Tsinghua University, Shenzhen, China

**Keywords:** short-video platform, livestream, health content, panel data, performance

## Abstract

**Introduction:**

Short-video platforms have demonstrated vast potential for health education. To meet diverse user requirements, many short-video platforms have integrated livestreaming functionalities. This integration presents challenges for health content creators in formulating effective performance strategies, including decisions about which format to use (short video or livestream) and what type of content to produce. This study utilizes panel data from a prominent short-video platform in China to empirically investigate the impact of different forms and content characteristics on the performance of health content creators.

**Methods:**

We conducted an empirical analysis using panel data obtained from a leading short-video platform in China. Our analysis focused on understanding how the behaviors associated with short videos and livestreaming impact the performance of health content creators. We examined form-level differences, analyzing the distinct roles of short video and livestreaming behaviors. Additionally, we explored content-level characteristics, investigating the effects of content coverage, health knowledge content, and advertising content on both short-term and long-term performance. The moderation effects of the creator’s occupation and certification type were also analyzed.

**Results:**

Our form-level analysis revealed that health creators’ behaviors in short videos and livestreaming play distinct roles in their performance. Livestreaming behaviors resulted in short-term economic returns, while short video behaviors had a more significant effect on follow-ups, which are often viewed as long-term, more sustainable performance indicators. Content-level analysis showed that content coverage and health knowledge content enhance long-term performance but do not increase short-term performance. Conversely, advertising content was found to be essential for securing short-term financial income. The study also identified that the creator’s occupation and certification type moderate the impact of content on performance.

**Conclusion:**

This study integrates two media forms (short video and livestream), providing direct insights into the performance of health content creators in the realm of health education. Health content creators need to strategically balance their use of short videos and livestreaming to optimize both short-term and long-term performance outcomes. Specifically, increasing content coverage and health knowledge can enhance long-term engagement, while incorporating advertising content is crucial for immediate financial gains.

## 1 Introduction

According to the 52 nd Statistical Report on China Internet Development, there were 1.026 billion users in the short-video platform by June 2023, with over 95.2% of netizens in the short-video field (1). The short-video platform serves as a convenient medium for the public to disseminate information based on its large community of users. In the health field, the public is accustomed to seeking health information (2, 3) and sharing personal health experiences via short-video platforms. The health information disclosures from celebrities can be a powerful source for health education (4). The short videos or livestreams produced by health content creators can help meet users’ health information demands (5), provide emotional support (6), and improve people’s health literacy (7). Therefore, many health celebrities and experts have played an opinion leadership role in it and have gained a large number of followers, sometimes up to millions.

However, in the actual competitive environment, health content creators are not all lucky. Many of them face the survival dilemma of limiting streaming, delaying updates, or even stopping updates. A short-video platform survey data shows that the operation data of health short videos is lower than the overall average level of the platform, and the feedback data (e.g., likes, comments, and reposts) obtained by health creators is also low (8). For instance, when creators struggle to generate sufficient economic returns, they find it challenging to cover the production costs of content and the operational expenses of their accounts. Alternatively, content creators may encounter challenges in establishing a loyal fan base, as well as obtaining suboptimal interactive data for their videos or livestreams. These factors can ultimately result in a decrease in their creative motivation (9). In the long term, as the quality of content and the quantity of content are affected, users’ health information needs cannot be met effectively. For the platform, it is also difficult to achieve commercial success. Therefore, how to promote the monetization and consolidation of traffic for e-health content, and to enhance the long-term and short-term performance of health content creators is a crucial question.

Researchers mainly focus on business strategies at the platform level to discuss the business ecosystem in a competitive two-sided market (10–13). Literature has discussed how the platform is governed under the ecosystem to capture the value and create performance from a macro perspective (14–16). However, concerning the interaction between health creators and viewers on short video sharing platforms, there is limited literature on empirical research exploring creators’ performance formation mechanism driven by the network effect from a micro perspective, especially for short video sharing platforms and some other two-sided markets. Although previous research has focus on the features of short videos or livestreams concerning creators’ marketing (17, 18), there is still a limited amount of research in the health domain. Moreover, this research often fails to comprehensively consider these two distinct forms of content creation. Therefore, our study aims to fill this research gap by answering the following research questions:

**RQ1**: *How does the creation form of digital health content affect the performance of health creators?*

**RQ2**: *How do the content characteristics of digital health content affect the performance of health creators?*

**RQ3**: *How do the creator characteristics affect the performance of health creators?*

In this research, we integrate two different media forms (short video and livestream) data and established theoretical frameworks to examine the effect of health content’s forms and content characteristics on the creators’ performance. By identifying the trends in the data, we aim to explore how health content creators with different identities can attract more followers or benefit financially more via their health content, and eventually receive long-term and short-term performances.

The rest of this study is organized as follows. In the second part, following the introduction, we will review the previous research on short video platforms and creator performance. In the third part, we present the hypothesis of this research, which is based on existing theories and research. In the fourth section, we analyze the research data and experimental variables, and use the fixed effects model to conduct an empirical study on the proposed hypothesis. In Parts V and VI, we present and test the results of our analysis and discuss key findings. Finally, we will discuss our results, propose theoretical and practical implications, consider the limitations of the study, and identify future research directions.

## 2 Literature review

### 2.1 Short-video platform and health content

Short-form mobile videos are video applications with functions of short video shooting, editing, uploading, and sharing (19). The short-video format requires users to express their opinions in a fast and engaging manner, while the casual nature and ability to interact with the audience allow for more personal and authentic communication (20). These characteristics allow the short-video platforms have rapidly expand worldwide. Platforms like Douyin and Kuaishou in China boast hundreds of millions of users. In recent years, the impact of COVID-19 has also prompted short-video platforms, such as TikTok, to show their potential in disseminating important health messages (2, 3). The user group that obtains health information through short-video platforms has become more extensive. The platform is particularly popular among teenagers, who may be receptive to receiving health information through it (21). Leveraging this vast user base, short-video platforms provide a convenient platform for health information communication (6).

According to previous research (22), visually rich health information has the advantage of attracting the audience’s attention and explaining key points. Therefore, short-video platforms exhibit unprecedented potential in the digital health domain, becoming a primary avenue for users to address health-related issues. Authoritative medical professionals can utilize online platforms to share practical experiences and professional knowledge, adopting a new role in fulfilling a mission to heal. Compared to traditional sources like search engines and online health communities, short video applications provide users with a more engaging experience (6). Particularly through short videos or livestreams, health information providers, disseminators, and users can engage more directly, fostering enhanced communication possibilities.

### 2.2 The health content and user’s interaction

In short-video platforms, short videos and livestreams serve as two main formats for health communication (18, 23). Previous research has primarily focused on one of them, especially in the medical and health domain, where studies concentrated on health short videos (6, 24). Studies regarding professionally generated content and user-generated content have examined information quality in disease education short videos (25–27) and preferences for health-related short videos (28), respectively. User-generated content studies usually focus on users’ intent to obtain health information (29) and their perceived usefulness of information (30). Livestreams have received less attention, with some scholars focusing on health live courses and their online dissemination effects (31). Others have studied doctors’ personal livestreams on third-party platforms, investigating factors influencing the popularity of medical professional livestreams (32). Previous research has focused solely on one form, either short videos or livestreams, exploring the interactive relationship between viewers and creators (33–37). However, our study integrates these aspects, providing a comprehensive understanding of the unique roles they play in health content production.

Researchers mainly focus on business strategies at the platform level to discuss the business ecosystem in a competitive two-sided market (10–13). Literature has discussed how the platform is governed under the ecosystem to capture the value and create performance from a macro perspective (14–16). However, concerning the interaction between health creators and viewers on short-video platforms, there is limited literature on empirical research exploring creators’ performance formation mechanism driven by the network effect from a micro perspective, especially for short-video platforms and some other two-sided markets. Although previous research has focused on the features of short videos or livestreams concerning creators’ marketing (17, 18), there is still a limited amount of research in the health domain. Table 1 shows how our study is positioned in the existing literatures.

**Table 1:** Existing literatures and our study.

Research Perspective	Research Content		Literatures	Our Study
Creator perspective	Short videos	Information quality assessment of short health videos	(25–27)	This study focuses on the interaction between creators and users, while considering the two forms of content creation, short video and live streaming, to fill the gap in related fields
		Explore user preferences for short video content	(28)	
	Livestreams	Explore the communication effect of live health course in public health field	(31)	
		Analyze the performance impact of doctors’ personal live streaming in third-party platforms	(32)	
Platform perspective		Explore the performance formation mechanism of short video platform under the competitive two-sided market	(14–16)	

### 2.3 Creator performance on short-video platforms

In the context of social media, performance can specifically be the volume of orders on online platforms (38), the quality of information communication and its effect on work efficiency (39), satisfaction, loyalty, and retention rate of corporate customers (40), and people’s perceived satisfaction in managing customer relationship (41). The performance indicators on short-video platforms, characterized by video information stream, may be slightly different from those on traditional social media. Short-video platforms represent a typical two-sided market, with multiple user groups that can provide benefits to one another (42).

In the health communication field, the “goods” provided by health content creators are health information of health content (short videos and livestreams). The feedback provided by viewers includes actions such as watching, liking, following, sharing, commenting, and purchasing products (43). Besides, some scholars also apply product diversity, visual effects, interaction, and duration to indicate creators’ performances in their livestreams (44). In this study, the transactions (orders and sales) and accumulated traffic (increment of followers) generated are of significant value to creators. We consider these as the short-term and long-term performance of creators, respectively. For health content creators, the short-term and long-term performance above can serve as the incentive to motivate them to engage persistently in content production and health education (9).

## 3 Hypotheses and research framework

The number of short videos and livestreams may impact the performance formation of creators. Some scholars have found that the volume of information published on online media can accelerate the spread of information to some extent (45). This phenomenon is likely to occur on short-video platforms as well. During a specific period, creators who publish more short videos and livestreams, updating more frequently, are more likely to be recognized as “active accounts” by the platform algorithm (46). Due to the recommendation mechanism of the algorithm, these creators who frequently publish short videos or livestreams are more likely to be recommended to more users (47). It may result in increased visibility, such as livestream views, or more opportunities for interaction, such as comments, likes, and shares. These can represent the popularity of short videos and livestreams. This exposure and interaction act as intuitive signals of sociality and social influence (48). The popularity of short videos and livestreams may attract viewers to provide further feedback.

Short videos are typically constrained to a duration of 5 min or less, featuring the characteristic of being short and concise (49). It enables viewers to quickly perceive the richness of content within the creator’s account in a short amount of time. This sense of immediacy can effectively promote the conversion of potential users. Stimulate the impulse for sustained attention, and ultimately lead to follower acquisition. Simultaneously, the attributes of easy dissemination and sharing of short videos can expand the creator’s influence, attracting a larger and more diverse fan base.

Different from short videos, livestreams have a longer duration, allowing for real-time interaction. Viewers can directly communicate with the creator and ask questions, Livestreams provide a sense of direct, synchronous communication (50). Frequent instant interactions can establish a deeper emotional connection between users and creators, thereby increasing users’ trust in the creator (4). The real-time nature of livestreams also allows products to be showcased in real-time within the livestream, enabling viewers to see the product’s usage, features, and effects. This intuitive demonstration helps increase the attractiveness of the product and the desire to purchase it. Besides, the active engagement of users in the sales process can enhance customers’ purchase intention (51), by increasing the perceived value or reducing perceived costs (52, 53). In other words, creators can receive higher financial returns for creators, such as increased orders and sales. Therefore, we propose Hypothesis 1a, 1b and 1c.

*H1a:* The number of short videos has a positive impact on creators’ long-term performance.

*H1b:* The number of livestreams has a positive impact on creators’ short-term performance.

*H1c:* The popularity of short videos and livestreams plays a mediating role in the respective performance formation processes.

Additionally, content features are also crucial factors that influence the performance of creators. Different content features may lead to different effects. In the process of information dissemination, information quality is a key factor influencing its effectiveness (54). Firstly, content richness and usefulness are critical factors in attracting user attention (55). The more coverage of digital health content, the easier it is to attract viewers of different types. It means the diversity of followers will be increased. Viewers may follow creators due to their interest in various topics, thereby expanding the creator’s viewer base. However, compared to followers’ attraction, the success of product promotion relies more on the purchasing demands and interests of the target audience. Overloading information from different themes may lead to viewers’ attention being dispersed, reducing the precision of product promotion (56, 57). Therefore, we propose hypothesis H2a:

*H2a:* The coverage of digital health content positively influences the long-term performance of creators.

Moreover, the determining factor for sustained engagement still largely depends on the quality of each video. In the electronic commerce platform, consumers’ purchasing decisions on e-commerce platforms are affected by the valence of the information received (58). TikTok users may initially engage more with micro-videos related to popular topics (59). For the viewers of digital health content, the usefulness of information is a crucial criterion for assessing its quality. Knowledge-based content often has a higher level of information, which helps to establish the creator’s professional image in the health field (60). It can enhance viewers’ trust to the creator, thereby they are more likely to engage, participate, and become loyal followers. However, for potential consumers of health products, the primary factor influencing their purchasing behaviour is the product’s usefulness (61). These viewers’ attention is primarily directed towards the product itself. Advertising health content typically showcases vibrant and user-friendly characteristics, enabling the rapid conveyance of product information and arousing consumer desires. Additionally, advertisements often create a relaxed and enjoyable atmosphere. The viewers’ positive interaction experience may prompt their purchasing behaviour. Therefore, we propose hypotheses H2b and H2c.

*H2b:* Knowledge health content positively influences the long-term performance of creators.

*H2c:* Advertisement health content positively influences the short-term performance of creators.

Lastly, we consider the information source—creator heterogeneity. The source of the message was found to have significant effects on perceptions of source credibility and message effectiveness (62). The participants perceived the doctor source to be more credible than the peer source and perceived the videos from the doctors to be more effective (63). A creator who is a chief physician in a hospital may gain more trust from viewers on medical educational videos they posted, thus encouraging more following behaviours. Scholars have found that the image features of doctors in online environments significantly impact viewers’ engagement. The doctors wearing work uniforms can convey a signal of professionalism, making it easier to gain the trust of patients in online settings (64). In the context of short-video platforms, creators holding the title of chief physician may achieve greater trust in their audience regarding medical knowledge-based short videos in related fields, thereby promoting audience engagement.

Meanwhile, creators’ authentication types may also influence the interaction between them and users. For instance, compared with creators without authentication, experts with a yellow “V” mark may find it easier to post works that can get users’ trust. In a word, creators’ characteristics may moderate short video popularity and viewers’ following and purchasing behaviours. For example, researchers have found that creators’ gender and occupation can moderate sales in livestreams (65). Meanwhile, an identity like an internet celebrity or opinion leader can also moderate viewers’ purchasing in transactions (35). Besides, studies also suggest that creators’ age, gender, credibility, and income can moderate the influence of fans’ following behaviours and other participation on their purchasing intention (36). Therefore, we can draw a hypothesis as follows:

*H3:* The personal characteristics of health content creators can play a moderating role in the performance formation process.

**Figure 1:**
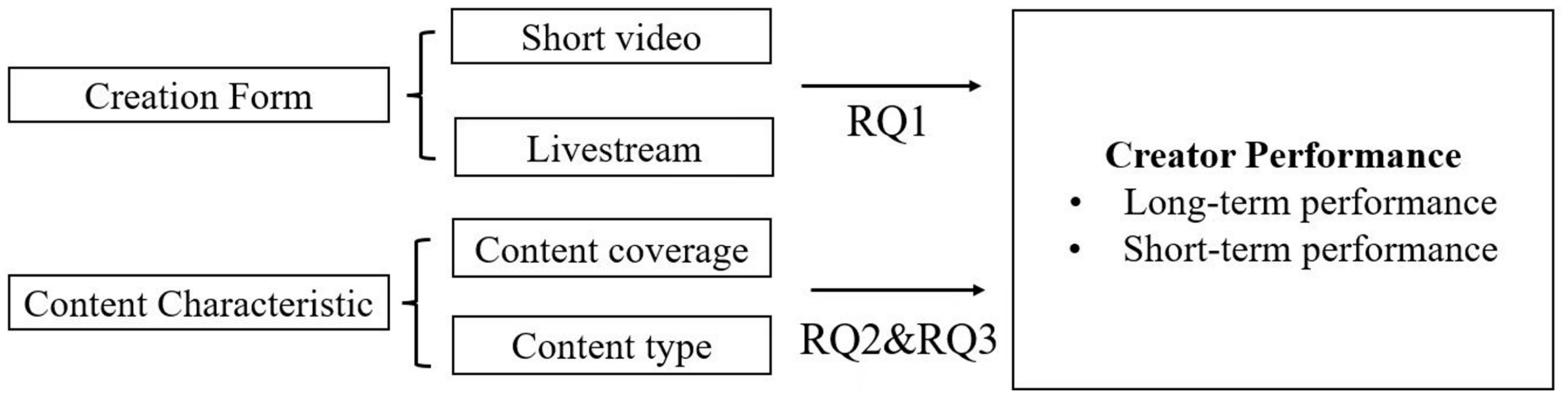
Research framework.

The framework of this study is shown in Figure 1.

## 4 Methods

### 4.1 Data collection

**Figure 2:**
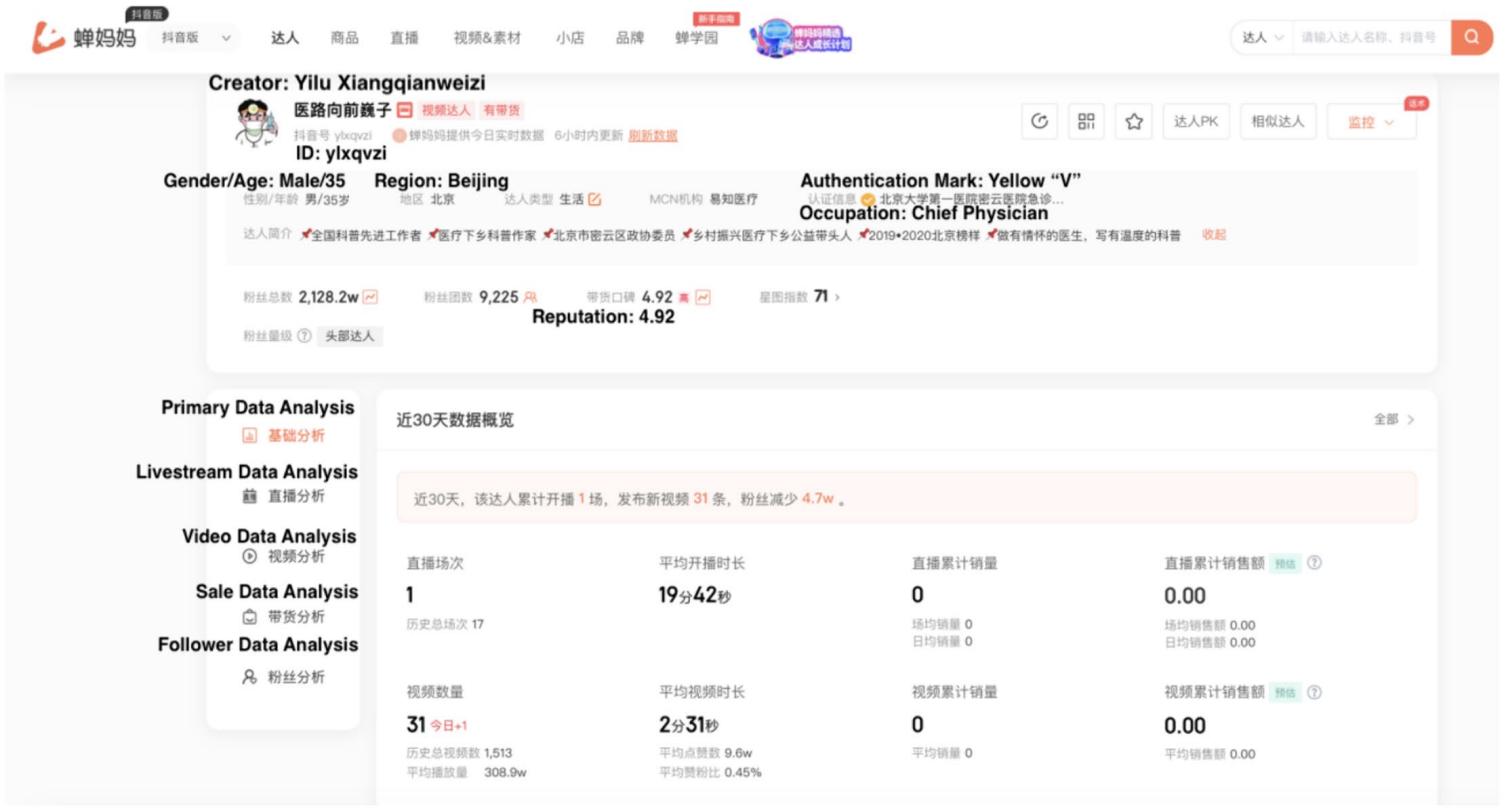
The creator page of Chanmama platform.

We selected the Douyin platform as our data source in this study. The data was collected from the Chanmama E-commerce Data Analysis Platform.^1^ This platform can be used to monitor the operation of accounts on Douyin and provide historical data to facilitate review. For example, we can browse the homepage of a health creator’s account “Yilu Xiangqianweizi” on Douyin. It’s shown in Figure 2. The platform provides the creator’s data, including ID, gender, age, occupation, reputation, and authentication type; livestream data, including the number of posted livestreams, views, the volume of orders, and sales; short video data, including the number of videos, likes, comments, and reposts; follower data, including the number of followers and its increment during a certain period. There are three types of health creators on Douyin: those with a yellow “V” mark, who are qualified experts like doctors and public health workers; blue “V” creators, usually representing companies; and creators without a mark, who are unverified individuals.

We wrote a Python web crawler program to collect our data from August 22nd, 2021 to February 10th, 2022. Our membership allows us to collect the top 300 creators’ data in the health ranking list. We consider every 7 days and 1 month as a period, resulting in a total of 25 week-periods and 6 month-periods, respectively.

### 4.2 Procedures and variables

Among the collected 300 creators’ data, 172 of those samples were excluded because they had no video or livestream updates for more than two consecutive weeks. We have checked that these creators did not carry out transactions on Douyin. Besides, 5 creators did not present their gender. Therefore, we filtered them and the remaining 123 creators were incorporated into our sample to analyze. The description of the sample is shown in Table 2. We generated two dummy variables for the creators’ authentication type. Meanwhile, we also normalized the number of likes, comments, and reposts in short videos and added them up to indicate the video popularity comprehensively. To alleviate the impact of heteroscedasticity on model estimation, the logarithm of absolute number was taken on variables including the number of videos, livestreams, new followers, livestream views, the volume of orders, and sales.

**Table 2:** Description of creators’ characteristics.

Discrete Variable	Num. (Percentage)
Gender	
Male	73 (59.35%)
Female	50 (40.65%)
Age	
20 ~ 29 years old	10 (8.13%)
30 ~ 39 years old	35 (28.46%)
40 ~ 49 years old	12 (9.76%)
50 ~ 59 years old	11 (8.94%)
60 years old and above	4 (3.25%)
NULL	51 (41.46%)
Occupation	
Non-doctor	75 (60.98%)
Doctor	48 (39.02%)
Authentication Type	
Yellow “V”	63 (51.22%)
Blue “V”	7 (5.69%)
No “V”	53 (43.09%)

To gain a more in-depth understanding of the thematic features of digital health content on short-video platforms, we conducted further classification of the short videos and livestreams published by creators. Drawing on existing research classification methodologies and aligning with the objectives of this study, we initially categorized health content on short-video platforms into four main types: Knowledge content (Clinical medicine content and Health management content), Advertising content, and Others. The key terms for text annotation are presented in Table 3.

**Table 3:** The coding of content type.

Content type	Definition	Examples
Knowledge Content	Clinical medicine content Content that is directly relevant to clinical practices, such as disease diagnosis and treatment, and medical examinations.	“Get to Know Fatty Liver in 3 Minutes.”
		“How to Understand Blood Routine Reports.”
		“Methods and Precautions for Using Antibiotics.”
	Health management content Content that focuses more on promoting a healthy lifestyle and practices, such as diet, exercise and psychological adjustment.	“How to have a balanced breakfast?”
		“For Friends with Shoulder Pain, Try These Exercises When You Have Time.”
		“Staring at the Phone Every Day, Eyes Feeling Dry and Tired? Here Are a Few Methods to Help You.”
Advertising Content	Content that clearly reflects marketing intentions, such as health product name, product branding and online course invitations.	“Bottle for Six Bottles, Affordable Deep-Sea Fish Oil for Everyone.”
		▪ “Effective Maintenance for Stomach Problems #XX Brand Probiotics.”
		“For Scientific and Healthy Weight Loss, My Livestream is the Right Place.”

Our primary focus for coding was on the titles of short videos and livestreams (including topic tags). The content type of health content is obtained by three researchers in related fields by marking the texts. Firstly, to ensure sample representativeness, we randomly selected 1,000 titles of short videos and livestreams as the initial marker sample. Three researchers individually labeled the knowledge content (Scientific Disease Knowledge and Lifestyle Health Management) and advertising content. For cases with discrepancies, the researchers engaged in discussions and ultimately reached a consensus. Subsequently, the researchers completed the labeling of the remaining sample titles, with each label representing a specific content type. Through comparisons, 86% of the samples were marked in agreement. Finally, we statistically determined the number of digital health content in different content types by transforming the coding results into corresponding continuous variables. And we calculated the standard deviation based on the distribution quantities to reflect the diversity of digital health content. In summary, variables incorporated in our models, as well as their meanings and descriptive statistics, are shown in Tables 4, 5. Finally, the preprocessed data was used to construct panel data.

**Table 4:** Descriptive statistics.

Variable	Observation	Mean	SD	Minimum	Maximum
Follow	3,075	5,778.60	30,148.33	−113654.0	465,799
Order	30,75	1,233.72	4,328.75	0	51,259
TR	3,075	59,227.13	197,844.81	0	3,342,097
Like	3,075	107,534.38	231,654.75	0	3,967,063
Comment	3,075	4,770.37	45,349.27	0	975,302
Share	3,075	20,577.96	345,913.53	0	10,122,040
PV	3,075	96,818.67	345,913.53	0	10,122,040
Video	3,075	6.18	6.16	0	65
Livestream	3,075	3.14	5.30	0	44
SciContent	738	10.16	17.67	0	202
DietContent	738	8.99	18.92	0	202
AdvContent	738	2.57	5.15	0	50
ContentCoverage	738	6.57	7.78	0	75.28

**Table 5:** Description of variables.

Variable	Name	Meaning
*ln(Video_it_)*	Num. of Short Videos	Number of short videos posted by creator *i* in period *t*
*ln(Livestream_it_)*	Num. of Livestreams	Number of livestreams posted by creator *i* in period *t*
*Popularity_it_*	Video Popularity	Video popularity of creator *i* in period *t*
*ln(SciContent_it_)*	Num. of Science content	Num. of Science content by creator *i* in period *t*
*ln(DietContentit)*	Num. of Diet content	Num. of Diet content by creator *i* in period *t*
*ln(AdvContent_it_)*	Num. of Advertisement content	Num. of Advertisement content by creator *i* in period *t*
*ContentCoverage_it_*	Health content coverage	The standard deviation of the number of different health content of creator *i* in period *t*
*ln(PV_it_)*	Livestream Views	Livestream views of creator *i* in period *t*
*ln(Follow_it_)*	Increment of Followers	Increment of followers of creator *i* in period t
*ln(Order_it_)*	Volume of Orders	Volume of orders in livestreams of creator *i* in period *t*
*ln(TR_it_)*	Sales	Sales in livestreams of creator *i* in period *t*
*Gender_i_*	Gender	Gender of creator *i*
*Age_i_*	Age	Age of creator *i*
*Occupation_i_*	Occupation	Occupation of creator *i*
*Type_1i_*	Authentication Type 1	The dummy variable 1 of creator i’s authentication type
*Type_2i_*	Authentication Type 2	The dummy variable 2 of creator i’s authentication type

### 4.3 Analytical strategies

This study applied panel data models to conduct the empirical analysis. To determine whether the fixed or random effect model is appropriate for our research, we conducted the Hausman Test in Table 6. It is tested that the *p* values on most explanatory variables are below 0.05, meaning that a fixed effect model is necessary. Besides, to control the impact of individual heterogeneity and common time trend on the model estimation, we developed fixed effect models and controlled the entity and time fixed effect simultaneously. And then we used StataSE 15.0 to conduct our analysis.

**Table 6:** Hausman test results.

Explained variable	Hausman test
ln(Follow_it_)	6.00
Popularity_it_	10.78^*^
ln(PV_it_)	7.87^*^
ln(Order_it_)	42.30^***^
ln(TR_it_)	33.61^***^

*p < 0.05; ***p < 0.001.

For hypotheses H1a and H1b, we developed a TWFE model as Equation 1, where *X_it_*. represents the number of short videos and livestreams, and *Y_it_*. denotes the increment of followers, volume of orders, and sales in each period. *α_i_* and *γ_t_* denote controlled entity and time-fixed effects, respectively. *β_j_* is the coefficient to be estimated, while *μ_it_* indicates the stochastic error.

(1) $$Y_{it}=\alpha_{i}+\gamma_{t}+\sum \beta_{j}\cdot \ln\left(X_{it}\right)+\mu_{it}\;$$

$$Y_{it}={\left(\;\ln\left(Follow_{it}\right),\ln\left(Order_{it}\right),\ln\left(TR_{it}\right)\right)}^{T}$$

$$X_{it}=\left(\;Video_{it},Livestream_{it}\;\right)$$

For hypotheses H1c, we employed a TWFE model as Equation 2. In this model, *M_it_* indicates the popularity of short video or livestream. The other coefficients are similar to Equation 1.

(2) $$Y_{it}^{’}=\alpha_{i}^{’}+\gamma_{t}^{’}+\sum \beta {’}_{j}\cdot \ln\left(X_{it}\right)+\beta^{’}\cdot M_{it}+\mu_{it}^{’}$$

$$Y_{it}^{’}={\left(\;\ln\left(Follow_{it}\right),\ln\left(Order_{it}\right),\ln\left(TR_{it}\right)\right)}^{T}$$

$$X{’}_{it}=\left(\;Video_{it},Livestream_{it}\;\right)$$

$$M_{it}=\left(\;Popularity_{it},PV_{it}\;\right)$$

For hypothesis H2, we developed the TWFE models as Equations 3, 4. In model Equations 3, *X_it_* and *X’_it_* represent, respectively, the coverage of content per period, and the number of science content, daily content, and advertisement content. Correspondingly, the increment of followers, volume of orders, and sales in the same period are explained variables, as shown in *Y_it_* and *Y’_it_*.

(3) $$Y_{it}=\theta_{i}+\lambda_{t}+\sum \beta_{j}\cdot \ln\left(X_{it}\right)+\mu_{it}$$

$$Y_{it}={\left(\;\ln\left(Follow_{it}\right),\ln\left(Order_{it}\right),\ln\left(TR_{it}\right)\right)}^{T}$$

$$X_{it}=\left(\;ContentCoverage_{it}\right)$$

(4) $$Y_{it}^{’}=\theta {’}_{i}+\lambda {’}_{t}+\sum \beta_{j}^{’}\cdot \ln\left(X_{it}^{’}\right)+\mu_{it}^{’}$$

$$Y_{it}^{’}={\left(\;\ln\left(Follow_{it}\right),\ln\left(Order_{it}\right),\ln\left(TR_{it}\right)\right)}^{T}$$

$$X{’}_{it}=\left(\;\ln\left(SciContent_{it}\right),\ln\left(DietContent_{it}\right),\ln\left(AdvContent_{it}\right)\;\right)$$

For hypothesis H3, two TWFE models were also developed as Equations 5, 6. The interaction terms between creators’ characteristics and various explanatory variables are incorporated into our models, respectively, to study the moderating effect. *CV_i_* is creators’ characteristic to be controlled.

(5) $$Y_{it}=\alpha_{i}+\gamma_{t}+\sum \beta_{j}\cdot \ln\left(X_{it}\right)+\sum \theta_{j}\cdot \;\ln\left(X_{it}\right)\cdot \;CV_{i}+\mu_{it}$$

$$Y_{it}={\left(\;\ln\left(Follow_{it}\right),\ln\left(Order_{it}\right),\ln\left(TR_{it}\right)\;\right)}^{T}$$

$$X_{it}={\left(\;ContentCoverage_{it}\right)}^{T}$$

$$CV_{i}=\left(\;Occupation_{i},Type_{1i},Type_{2i}\;\right)$$

(6) $$Y_{it}^{’}=\alpha_{i}^{’}+\gamma_{t}^{’}+\sum \beta_{m}\cdot \ln\left(X_{it}^{’}\right)+\sum \theta_{m}\cdot \;\ln\left(X_{it}^{’}\right)\cdot \;CV_{i}+\mu_{it}^{’}$$

$$Y_{it}^{’}={\left(\;\ln\left(Follow_{it}\right),\ln\left(Order_{it}\right),\ln\left(TR_{it}\right)\right)}^{T}$$

$$X_{it}^{’}={\left(\;\ln\left(SciContent_{it}\right),\ln\left(DietContent_{it}\right),\ln\left(AdvContent_{it}\right)\right)}^{T}$$

$$CV_{i}=\left(\;Occupation_{i},Type_{1i},Type_{2i}\;\right)$$

## 5 Results

### 5.1 The impact of creation form on creator performance

#### 5.1.1 The direct influence

For H1, we empirically tested the direct influence. The results are shown in Table 6. The results indicated that the number of short videos posted by creators can have a significant influence on the increment of followers (*β* = 0.025, *p* < 0.05) in the current period. However, we did not identify a similar impact on the growth of orders and sales in the current period. Meanwhile, the livestream behavior can also positively influence the growth of orders (*β* = 2.11, *p* < 0.01) and sales (*β* = 3.452, *p* < 0.01).

#### 5.1.2 Mediating effect of popularity

H1c examined the mediator effect of the video popularity and the livestream popularity. The results are summarized in Table 7. Specifically, video popularity can have a significantly positive influence on the increment of followers in the current period (*β* = 0.561, *p* < 0.01). According to columns 6 and 9 of Table 7, the popularity of livestreams also plays a positive mediating role in the number of livestreams and the growth of orders (*β* = 0.279, *p* < 0.01) and sales (*β* = 0.548, *p* < 0.01). Hence, we find that the efforts made by creators in short videos or livestreams will initially accumulate into data traffic, which will then translate into long-term or short-term performance. In a word, H1 is proven.

**Table 7:** The impact of creation forms.

Variable	ln(*Follow_it_*)			ln(*Order_it_*)			ln(*TR_it_*)		
	Direct	Mediator		Direct	Mediator		Direct	Mediator	
*ln(Video_it_)*	0.025^**^ (0.011)	0.001 (0.009)	0.025^**^ (0.011)	0.034 (0.070)	0.044 (0.071)	0.039 (0.069)	0.059 (0.108)	0.075 (0.110)	0.069 (0.105)
*ln(Livestream_it_)*	0.007 (0.005)	0.009 (0.006)	0.005 (0.011)	2.110^***^ (0.206)	2.109^***^ (0.206)	0.641^**^ (0.272)	3.452^***^ (0.315)	3.451^***^ (0.315)	0.566 (0.424)
*Popularity*		0.561^***^ (0.160)			−0.236 (0.185)			−0.391 (0.299)	
*lnPV*			0.000 (0.002)			0.279^***^ (0.046)			0.548^***^ (0.069)
Creator FE	Yes	Yes	Yes	Yes	Yes	Yes	Yes	Yes	Yes
Time FE	Yes	Yes	Yes	Yes	Yes	Yes	Yes	Yes	Yes
Observations	3,075	3,075	3,075	3,075	3,075	3,075	3,075	3,075	3,075
Adjusted *R*^2^	0.016	0.076	0.016	0.388	0.388	0.457	0.389	0.389	0.491

Standard errors are in parentheses. *p < 0.1; **p < 0.05; ***p < 0.01.

### 5.2 The impact of content characteristics on creator performance

#### 5.2.1 The impact of content coverage

Health content creators usually post a limited number of short videos or livestreams per week, usually ranging from 2 to 5. After subdividing the weekly data according to content type, we found that the number of videos under each subcategory (Science, Diet, Advertisement) would have more 0 values. To better observe the impact of specific theme characteristics on creators’ performance, we replaced the weekly data with the monthly data. The estimation results of H3 are shown in Table 7. We found that the wider the content coverage of short videos and livestreams, the greater the number of new followers attracted during the same period (*β* = 0.018, *p* < 0.05).

#### 5.2.2 The impact of content type

Additionally, we examined the influence of content type, specifically focusing on clinical medicine, health management, and advertising content. Tables 8, 9 showed that both the content related to clinical medicine and health management would have a positive impact on the followers’ increment. Specifically, when compared to the science content (*β* = 0.122, *p* < 0.01), the diet content (*β* = 0.178, *p* < 0.01) has a stronger positive impact on the creator’s long-term performance. This suggests that creators posting short videos related to daily health management are more conducive to attracting followers. However, there was no clear relationship between publishing advertising content and the increment of followers. The advertising content can increase the creators’ orders (*β* = 0.104, *p* < 0.01) and sales (*β* = 0.152, *p* < 0.01).

**Table 8:** The impact of content coverage.

Explanatory variable	Explained variable		
	ln(*Follow_it_*)	ln(*Order_it_*)	ln(*TR_it_*)
*ContentCoverage_it_*	0.018^**^ (0.007)	0.013 (0.017)	0.016 (0.029)
Creator FE	Yes	Yes	Yes
Time FE	Yes	Yes	Yes
Observations	738	738	738
Adjusted *R*^2^	0.071	0.023	0.019

Standard errors are in parentheses. *p < 0.1; **p < 0.05; ***p < 0.01.

**Table 9:** The impact of content type.

Explanatory variable	Explained variable		
	ln(*Follow_it_*)	ln(*Order_it_*)	ln(*TR_it_*)
*ln(SciContent_it_)*	0.122^***^ (0.040)	0.265 (0.213)	0.221 (0.371)
*ln(DietContent_it_)*	0.178^***^ (0.034)	−0.070 (0.094)	−0.113 (0.154)
*ln(AdvContent_it_)*	−0.002 (0.003)	0.104^***^ (0.028)	0.152^***^ (0.041)
Creator FE	Yes	Yes	Yes
Time FE	Yes	Yes	Yes
Observations	738	738	738
Adjusted *R*^2^	0.225	0.087	0.072

Standard errors are in parentheses. *p < 0.1; **p < 0.05; ***p < 0.01.

### 5.3 Moderating effect of creator characteristics

To further explore content creation strategies for different creators, we consider the characteristics of the creators. According to H3, the dual fixed effect model of individual and time-point was established to analyze the moderating effects of the characteristics of creators, including the occupation and certification type. Table 10 shows the estimation results.

**Table 10:** Moderating Effect of creators’ characteristics.

	ln(*Follow_it_*)	ln(*Order_it_*)	ln(*TR_it_*)	ln(*Follow_it_*)	ln(Orderit)	ln(*TR_it_*)
Explanatory variable	Model 1			Model 2		
*ContentCoverage_it_*	0.018^**^ (0.007)	0.013 (0.017)	0.016 (0.029)	−0.002 (0.005)	-	-
*Occupation_i_*	-	-	-	-	-	-
ContentCoverage_it_* Occupation_i_	-	-	-	0.030^***^ (0.008)	-	-
Creator FE	Yes	Yes	Yes	Yes	-	-
Time FE	Yes	Yes	Yes	Yes	-	-
Observations	738	738	738	738	-	-
Adjusted *R*^2^	0.071	0.023	0.019	0.107	-	-
**Explanatory variable**	**Model 3**			**Model 4**		
*ln(SciContent_it_)*	0.067^***^ (0.029)	0.148 (0.223)	0.138 (0.357)	0.018 (0.019)	0.343 (0.320)	0.472 (0.501)
*ln(DietContent_it_)*	0.132^***^ (0.029)	−0.068 (0.087)	−0.110 (0.129)	0.058^***^ (0.017)	−0.030 (0.131)	−0.038 (0.187)
*ln(AdvContent_it_)*	−0.019 (0.015)	0.347^***^ (0.133)	0.511^***^ (0.202)	−0.024 (0.018)	0.092^***^ (0.025)	0.221 (0.247)
*Occupation_i_*	-	-	-	-	-	-
ln(SciContent_it_)*Occupation_i_	-	-	-	0.139^**^ (0.057)	-	-
ln(DietContent_it_)*Occupation_i_	-	-	-	0.173^***^ (0.049)	-	-
ln(AdvContent_it_)*Occupation_i_	-	-	-	-	0.335 (0.255)	0.817^**^ (0.385)
Creator FE	Yes	Yes	Yes	Yes	Yes	Yes
Time FE	Yes	Yes	Yes	Yes	Yes	Yes
Observations	738	738	738	738	738	738
Adjusted *R*^2^	0.152	0.041	0.033	0.221	0.049	0.046
**Explanatory variable**	**Model 5**			**Model 6**		
*ContentCoverage*	0.018^**^ (0.007)	0.013 (0.017)	0.016 (0.029)	0.025^***^ (0.007)	-	-
*Type1_i_*	-	-	-	-	-	-
*Type2_i_*	-	-	-	-	-	-
ContentCoverage_it_* Type1_i_	-	-	-	−0.004 (0.015)	-	-
ContentCoverage_it_* Type2_i_	-	-	-	−0.033^***^ (0.009)	-	-
Creator FE	Yes	Yes	Yes	Yes	-	-
Time FE	Yes	Yes	Yes	Yes	-	-
Observations	738	738	738	738	-	-
Adjusted *R*^2^	0.152	0.041	0.033	0.101	-	-
**Explanatory variable**	**Model 7**			**Model 8**		
*ln(SciContent_it_)*	0.067^***^ (0.029)	0.148 (0.223)	0.138 (0.357)	0.148^***^ (0.047)	−0.251 (0.264)	−0.625 (0.449)
*ln(DietContent_it_)*	0.132^***^ (0.029)	−0.068 (0.087)	−0.110 (0.129)	0.058^***^ (0.017)	−0.028^**^ (0.014)	−0.159 (0.142)
*ln(AdvContent_it_)*	−0.019 (0.015)	0.347^***^ (0.133)	0.511^***^ (0.202)	−0.024 (0.018)	0.115^**^ (0.053)	0.649^**^ (0.278)
*Type1_i_*	-	-	-	-	-	-
*Type2_i_*	-	-	-	-	-	-
ln(SciContent_it_)* Type1_i_	-	-	-	−0.153^***^ (0.050)	-	-
ln(SciContent_it_)* Type2_i_	-	-	-	−0.153^***^ (0.050)	-	-
ln(DietContent_it_)* Type1_i_	-	-	-	−0.115^**^ (0.045)	-	-
ln(DietContent_it_)* Type2_i_	-	-	-	−0.116^**^ (0.046)	-	-
ln(AdvContent_it_)* Type1_i_	-	-	-	-	0.145 (0.846)	−0.257 (1.512)
ln(AdvContent_it_)*Type2_i_	-	-	-	-	−0.086 (0.270)	−0.333 (0.405)
Creator FE	Yes	Yes	Yes	Yes	Yes	Yes
Time FE	Yes	Yes	Yes	Yes	Yes	Yes
Observations	738	738	738	738	738	738
Adjusted *R*^2^	0.225	0.087	0.072	0.193	0.055	0.051

Standard errors are in parentheses. *p < 0.1; **p < 0.05; ***p < 0.01.

Specifically, compared with non-doctor (*Occupationi* = 0), the identity as doctor (*Occupationi* = 1) has strengthened the influence of content coverage on the followers’ increment (*β* = 0.030, *p* < 0.01). Regarding content types, when compared to non-doctor creators, doctors who share knowledge content, including disease education (*β* = 0.139, *p* < 0.05) and health management (*β* = 0.173, *p* < 0.01), can attract more followers. Similarly, advertising content published by doctors is more likely to result in their sales (*β* = 0.817, *p* < 0.05). We found that on short-video platforms, the occupation serves as an effective signal, making it easier for creators to achieve performance.

We also found that compared to yellow “V” (*Type_1i_* = 0, *Type2i* = 0) and blue “V” (*Type_1i_* = 1, *Type2i* = 0), creators who have no authentication mark (*Type_1i_* = 0, *Type2i* = 1) can weaken the influence of content coverage on the increment of followers (*β* = −0.033, *p* < 0.01). It means that health creators without authentication may have less advantage in spreading their works, disseminating health information, and attracting followers. What’s more, compared to yellow “V,” creators who have a blue “V” and no authentication mark can weaken the influence of knowledge content on the increment of followers. It denotes that people may prefer knowledge content released by certified experts.

All in all, occupation and authentication type can significantly moderate the impact of content characteristics on creators’ long-term and short-term performance. Thus, H3 is proved.

## 6 Robustness check

### 6.1 The influence of the blue “V” identity

Table 2 shows that there are 7 creators with blue “V” in our sample. They mainly represent the official or company. However, we have noted that among the works posted by them, many are not related to health. And the type of these works is generally more comprehensive. For example, the account “Yuan Hetang” is operated by Beijing Yuanhe Yuyue Culture Communication Co., Ltd. This account not only releases work about traditional Chinese medicine, but often introduces the culture of Chinese incense (see the video “Meditate with Chinese incense and make an incense seal together”). Meanwhile, this account also posts some other cultural videos, such as traditional wine-making technologies (see the video “Green plum wine-collect the spring in a jar and open it at the end of summer” and “There is pure spring water in mountains and let us go to make wine”). Besides, the account “Chengcheng Xinshe” operated by Chengcheng Xinshe Tea Ceremony Studio mainly releases works related to self-cultivation, among which are usually about sentiment and traditional culture (see the video “When climbing a mountain where will be many paths; which path you take is your chance...” and “The custom of the Tomb Sweeping Day—go for worshiping ancestors”). These videos may not be related to health, so we filtered these 7 creators from our sample to test whether our models are still robust. The results are shown in Supplementary Tables 1–4. It can be seen that all coefficients have changed slightly, but the sign and significance of our interested variables are stable. Therefore, the results and findings we have identified are robust to our filtered sample.

### 6.2 The influence of common time trend

We have developed fixed effect models and controlled the entity and time-fixed effect simultaneously. Now we consider relaxing this restriction and just control the entity fixed effect to test the influence of common time trend on the robustness of our models, and the results are shown in Supplementary Tables 5–8. Reassuringly, the results of the robustness check are qualitatively consistent with the original results.

## 7 Discussion

### 7.1 Principal findings

This study applied TWFE models to analyze 25 weekly and 6 monthly data from 123 health content creators on a short-video platform. By employing comprehensive analysis, we studied the effect of creation forms and content characteristics on the health creator’s performance.

Firstly, the results showed that the creation form has different mechanisms of influence on the long-term and short-term performance of health creators (RQ1). On the one hand, creators’ efforts in short videos result in more follower engagement, contributing to long-term performance. This finding aligns with existing research on social media (66, 67). On the other hand, creators’ short-term economic performance is derived from their investment in livestreams. This could be due to the real-time interaction and the deeper engagement of livestreams. In this way, it’s easier to build trust with users, thereby prompting them to make purchase decisions.

Secondly, in terms of content characteristics, the wider the coverage of content in health creation (short video or livestream), and health creators can gain more followers’ attention (RQ2). This is consistent with previous research results, to some extent proving that users on short-video platforms have a high demand for information. Notably, content type has different impacts on creator performance (RQ2). Specifically, compared to specialized content, like clinical medicine, more lifestyle-oriented content such as diet management and emotional regulation is more conducive to attracting followers. A possible explanation is that, unlike traditional media like print, the information reception threshold for short videos is lower. Content with low knowledge acquisition costs and easy-to-understand popular science content is more effective in public health education. Another possible explanation is that the style of health management content is generally more relaxing and interesting, making them closer to users’ daily life. This can effectively meet users’ dual needs for the informative and entertaining aspects of health content, motivating them to stay engaged with subsequent content. In addition, only advertising-based content can increase short-term performance.

Third, the most intriguing finding was the interaction effect of creators’ characteristics and content characteristics for creators’ performance (RQ3). Specifically, a doctor’s professional identity is a reliable signal, and people are more willing to follow health content published by professional doctors. Due to unlike other general short videos, the reliability of the health knowledge disseminated by creators is a key factor in determining whether people are willing to follow someone in the long term. Furthermore, doctors outperform non-doctor creators in sales performance. This indicates that, not only on traditional social media platforms, but also on short-video platforms, a doctor’s identity has a natural competitive advantage. Consistent with previous findings (68), platform certification is also a crucial factor. As visible signals on short-video platforms, it can contribute to creators achieving more performance.

### 7.2 Research implications

#### 7.2.1 Theoretical implications

Several theoretical implications should be noted. The prominent contribution of this paper is taking an in-depth look at the format and content of health content. Previous research has focused solely on one format, either short videos or livestreams, exploring the interactive relationship between viewers and creators (33–37). However, our study comprehensively considered both short videos and livestreams, conducting integrated research. Meanwhile, the analysis revealed the impact mechanisms of different content creation forms on the performance of health creators. Specifically, livestreaming behaviors result in some short economic returns, while short video behaviors have a more significant effect on follow-ups, which are often viewed as a long-term, more sustainable performance. The literature in this field has been enriched and the interaction pattern between creators and viewers become clearer. Additionally, it categorizes the thematic content of health content, yielding more comprehensive research findings.

Moreover, this study selected health content creators as the focal point, enriching research related to health content creation. Previous research has mainly focused on the aspect of audience and content itself, such as information dissemination attributes of health short videos (69, 70). And some scholars focus on the assessment of the quality of health content (26, 54), exploring factors influencing the spread of health short videos. However, our study expands the scope to include the marketing attributes of health content, confirming the monetization potential of health short videos. It can enrich relevant theories and provide more practical guidance and suggestions for healthy content creation practice.

#### 7.2.2 Practical implications

Firstly, our research findings provide guidance for health content creators with different performance goals. As short-video platforms mature, more content producers are not confined to the development of a single form, whether it be short videos or livestreams. Our research confirms that short videos and livestreams have different performance mechanisms, which is practically significant. The results suggest that health creators need to balance their energy allocation between short videos and livestreams to seek diversified development. Additionally, in content selection, we provide corresponding guidance. For example, content richness helps attract more followers. While focusing on advertising content is more effective in practical terms when it comes to product promotion. This implies that health content creators need to clarify their account positioning and maintain the professionalism of content. In addition, to gain more followers, yellow and blue V creators should expand and enrich the topics of their videos as much as possible.

Secondly, our research findings offer guidance for different types of health content creators. Nowadays, short-video platforms, due to their inclusive and diversified development characteristics, are attracting more users, presenting a trend of diversification among content producers. Similarly, in the field of health education, the types of health content creators are becoming more diverse, with more people participating in health communication. Based on the personalized characteristics of creators, our research results provide guiding opinions. For example, creators with a medical background should publish more professional health content. By leveraging their advantage in attracting followers, they can play a greater role in health education. Yellow V creators can gain more recognition from fans by frequently publishing professional popular science and health management content.

Thirdly, this study also inspires platforms and users to make their efforts in health communication. For example, platforms should encourage creators to actively produce health knowledge content, fostering an active community of health creators. Additionally, urging professionals such as doctors and nursing staff to join the ranks of health content creators can leverage their professional advantages to disseminate higher-quality health knowledge and promote a mutually beneficial relationship between the platform and creators. Moreover, as ordinary users, actively engaging with creators and providing timely positive encouragement is crucial. This interaction helps maintain creators’ enthusiasm for content creation and serves as a key driver of performance conversion.

Lastly, metaphorical interpretation is also one of the important problems to be considered in practice. In the process of public health communication, short videos or live streams, as highly visual new media, inevitably involve metaphorical interpretations (71). Such metaphorical interpretations may affect the accuracy of information transmission from health content creators to the public. Therefore, content creators should primarily use clear and straightforward language and visual elements to avoid overly complex or abstract metaphors. Describing core concepts directly can help minimize ambiguity during information transmission. Platforms should also strengthen their review mechanisms to prevent the spread of misinformation and public health anxiety caused by information misinterpretation. Additionally, incorporating interactive elements in live streams or short videos, such as responding to audience questions or feedback, can address potential misunderstandings in real time. This not only strengthens audience engagement but also reduces information bias caused by metaphors.

### 7.3 Limitations and future research directions

However, there are still some limitations and potential future work. First, due to data constraints, we focused on the impact of different health content types but did not account for content quality. Future work can develop evaluation standards to assess how content quality affects creator performance. Secondly, as user data were not available, our analysis was limited to the creator’s perspective. Future studies could explore creator-user interactions to enhance the model through natural or quasi-experimental designs. Lastly, our methodology and research questions could be tested on more globalized social media platforms to expand the validity and generalizability of our findings.

## Data Availability

The raw data supporting the conclusions of this article will be made available by the authors, without undue reservation.

## Footnotes

^1^www.chanmama.com
