# Supplementary material for: How do health content creators perform well? An integration research of short video and livestream behaviors
Source: Front Public Health. 2024 Oct 2;12:1446247. doi: 10.3389/fpubh.2024.1446247 (PMC11480066; doi:10.3389/fpubh.2024.1446247)
Supplement: Supplementary file 1 [file Table_1.docx]

Supplementary Material

# Supplementary Tables

The results for the robustness checks are presented in Tables 1-8 below.

**1.1 The influence of blue “V” identity**

**Table 1** The Impact of Creation Forms (Blue “V” Filtered)

| **Variable** | **ln(*Follow_it_*)** | | | **ln(*Order_it_*)** | | | **ln(*TR_it_*)** | | |
| --- | --- | --- | --- | --- | --- | --- | --- | --- | --- |
|  | Direct | Mediator | | Direct | Mediator | | Direct | Mediator | |
| *ln(Video_it_)* | 0.026^**^  (0.012) | 0.001  (0.010) | 0.026^**^  (0.012) | 0.026  (0.074) | 0.036  (0.075) | 0.028  (0.073) | 0.066  (0.113) | 0.082  (0.116) | 0.068  (0.111) |
| *ln(Livestream_it_)* | 0.007  (0.006) | 0.009  (0.006) | 0.007  (0.011) | 2.150^***^  (0.205) | 2.150^***^  (0.205) | 0.775^**^  (0.248) | 3.489^***^  (0.305) | 3.487^***^  (0.308) | 0.792^**^  (0.385) |
| *Popularity* | - | 0.561^***^  (0.161) | - | - | -0.217  (0.186) | - | - | -0.367  (0.301) | - |
| *lnPV* | - | - | 0.000  (0.002) | - | - | 0.263^***^  (0.046) | - | - | 0.515^***^  (0.068) |
| Creator FE | Yes | Yes | Yes | Yes | Yes | Yes | Yes | Yes | Yes |
| Time FE | Yes | Yes | Yes | Yes | Yes | Yes | Yes | Yes | Yes |
| Observations | 2900 | 2900 | 2900 | 2900 | 2900 | 2900 | 2900 | 2900 | 2900 |
| Adjusted *R*^2^ | 0.016 | 0.076 | 0.016 | 0.426 | 0.388 | 0.490 | 0.422 | 0.426 | 0.517 |

Notes: standard errors are in parentheses. * *p* < 0.1; ** *p* < 0.05; *** *p* < 0.01.

**Table 2** The Impact of Content Coverage (Blue “V” Filtered)

| **Explanatory Variable** | **Explained Variable** | | |
| --- | --- | --- | --- |
|  | ln(*Follow_it_*) | ln(*Order_it_*) | ln(*TR_it_*) |
| *ContentCoverage_it_* | 0.018^**^  (0.007) | 0.013  (0.017) | 0.019  (0.029) |
| Creator FE | Yes | Yes | Yes |
| Time FE | Yes | Yes | Yes |
| Observations | 696 | 696 | 696 |
| Adjusted *R*^2^ | 0.071 | 0.026 | 0.021 |
| Notes: standard errors are in parentheses. * *p* < 0.1; ** *p* < 0.05; *** *p* < 0.01. | | | |

**Table 3** The Impact of Content Type (Blue “V” Filtered)

| **Explanatory Variable** | **Explained Variable** | | |
| --- | --- | --- | --- |
|  | ln(*Follow_it_*) | ln(*Order_it_*) | ln(*TR_it_*) |
| *ln(SciContent_it_)* | 0.076^**^  (0.030) | 0.163  (0.239) | 0.129  (0.385) |
| *ln(DietContent_it_)* | 0.136^*^  (0.030) | -0.015  (0.011) | -0.091  (0.131) |
| *ln(AdvContent_it_)* | -0.019  (0.016) | 0.346^**^  (0.135) | 0.516^**^  (0.205) |
| Creator FE | Yes | Yes | Yes |
| Time FE | Yes | Yes | Yes |
| Number of Users | 116 | 116 | 116 |
| Number of Periods | 6 | 6 | 6 |
| Observations | 696 | 696 | 696 |
| Adjusted *R*^2^ | 0.156 | 0.044 | 0.035 |
| Notes: standard errors are in parentheses. * *p* < 0.1; ** *p* < 0.05; *** *p* < 0.01. | | | |

**Table 4** Moderating Effect of Creators’ Characteristics (Blue “V” Filtered)

| **Explanatory Variable** | **Model 1** | | | **Model 2** | | |
| --- | --- | --- | --- | --- | --- | --- |
|  | ln(*Follow_it_*) | ln(*Order_it_*) | ln(*TR_it_*) | ln(*Follow_it_*) | ln(Orderit) | ln(*TR_it_*) |
| *ContentCoverage_it_* | 0.018^**^  (0.003) | 0.013  (0.018) | 0.016  (0.030) | -0.002  (0.005) | - | - |
| *Occupation_i_* | - | - | - | - | - | - |
| *ContentCoverage_it_* Occupation_i_* |  |  |  | 0.031^***^  (0.008) | - | - |
| Creator FE | Yes | Yes | Yes | Yes | - | - |
| Time FE | Yes | Yes | Yes | Yes | - | - |
| Observations | 696 | 696 | 696 | 696 | - | - |
| Adjusted *R*^2^ | 0.071 | 0.023 | 0.019 | 0.107 | - | - |
| **Explanatory Variable** | **Model 3** | | | **Model 4** | | |
|  | ln(*Follow_it_*) | ln(*Order_it_*) | ln(*TR_it_*) | ln(*Follow_it_*) | ln(Orderit) | ln(*TR_it_*) |
| *ln(SciContent_it_)* | 0.076^**^  (0.030) | 0.163  (0.239) | 0.129  (0.385) | 0.021  (0.030) | 0.398  (0.262) | 0.504  (0.373) |
| *ln(DietContent_it_)* | 0.136^*^  (0.030) | -0.015  (0.011) | -0.091  (0.131) | 0.058^***^  (0.001) | -0.015  (0.910) | -0.009  (0.964) |
| *ln(AdvContent_it_)* | -0.019  (0.016) | 0.346^**^  (0.135) | 0.516^**^  (0.205) | -0.024  (0.199) | 0.211  (0.226) | 0.213  (0.404) |
| *Occupation_i_* | - | - | - | - | - | - |
| *ln(SciContent_it_)*Occupation_i_* | - | - | - | 0.137^***^  (0.019) | - | - |
| *ln(DietContent_it_)*Occupation_i_* | - | - | - | 0.172^***^  (0.001) | - | - |
| *ln(AdvContent_it_)*Occupation_i_* | - | - | - | - | 0.353  (0.174) | 0.82^**^  (0.037) |
| Creator FE | Yes | Yes | Yes | Yes | Yes | Yes |
| Time FE | Yes | Yes | Yes | Yes | Yes | Yes |
| Observations | 696 | 696 | 696 | 696 | 696 | 696 |
| Adjusted *R*^2^ | 0.156 | 0.044 | 0.035 | 0.221 | 0.054 | 0.049 |
| **Explanatory Variable** | **Model 5** | | | **Model 6** | | |
|  | ln(*Follow_it_*) | ln(*Order_it_*) | ln(*TR_it_*) | ln(*Follow_it_*) | ln(Orderit) | ln(*TR_it_*) |
| *ContentCoverage_it_* | 0.018^**^  (0.003) | 0.013  (0.018) | 0.016  (0.030) | 0.025^***^  (0.007) | - | - |
| *Type1_i_* | - | - | - | - | - | - |
| *Type2_i_* | - | - | - | - | - | - |
| *ContentCoverage_it_* Type1_i_* | - | - | - | - | - | - |
| *ContentCoverage_it_* Type2_i_* | - | - | - | -0.033^***^  (0.009) | - | - |
| Creator FE | Yes | Yes | Yes | Yes | - | - |
| Time FE | Yes | Yes | Yes | Yes | - | - |
| Observations | 696 | 696 | 696 | 696 | - | - |
| Adjusted *R*^2^ | 0.071 | 0.023 | 0.019 | 0.101 | - | - |
| **Explanatory Variable** | **Model 7** | | | **Model 8** | | |
|  | ln(*Follow_it_*) | ln(*Order_it_*) | ln(*TR_it_*) | ln(*Follow_it_*) | ln(Orderit) | ln(*TR_it_*) |
| *ln(SciContent_it_)* | 0.076^**^  (0.030) | 0.163  (0.239) | 0.129  (0.385) | 0.148^***^  (0.047) | -0.253  (0.264) | -0.621  (0.448) |
| *ln(DietContent_it_)* | 0.136^*^  (0.030) | -0.015  (0.011) | -0.091  (0.131) | 0.173^***^  (0.040) | -0.028^**^  (0.014) | -0.159  (0.180) |
| *ln(AdvContent_it_)* | -0.019  (0.016) | 0.346^**^  (0.135) | 0.516^**^  (0.205) | 0.001  (0.022) | 0.377^**^  (0.173) | 0.652^***^  (0.277) |
| *Type1_i_* | - | - | - | - | - | - |
| *Type2_i_* | - | - | - | - | - | - |
| *ln(SciContent_it_)* Type1_i_* | - | - |  |  | - | - |
| *ln(SciContent_it_)* Type2_i_* | - | - |  | -0.153^***^  (0.050) | - | - |
| *ln(DietContent_it_)* Type1_i_* | - | - |  |  | - | - |
| *ln(DietContent_it_)* Type2_i_* | - | - |  | -0.116^**^  (0.046) | - | - |
| *ln(AdvContent_it_)* Type1_i_* | - | - | - | - | - | - |
| *ln(AdvContent_it_)*Type2_i_* | - | - | - | - | -0.092  (0.268) | -0.332  (0.404) |
| Creator FE | Yes | Yes | Yes | Yes | Yes | Yes |
| Time FE | Yes | Yes | Yes | Yes | Yes | Yes |
| Observations | 696 | 696 | 696 | 696 | 696 | 696 |
| Adjusted *R*^2^ | 0.156 | 0.044 | 0.035 | 0.193 | 0.060 | 0.054 |

Notes: standard errors are in parentheses. * *p* < 0.1; ** *p* < 0.05; *** *p* < 0.01.

**1.2 The influence of common time trend**

**Table 5** The Impact of Creation Forms (Common Time Trend Uncontrolled)

| **Variable** | **ln(*Follow_it_*)** | | | **ln(*Order_it_*)** | | | **ln(*TR_it_*)** | | |
| --- | --- | --- | --- | --- | --- | --- | --- | --- | --- |
|  | Direct | Mediator | | Direct | Mediator | | Direct | Mediator | |
| *ln(Video_it_)* | 0.025^**^  (0.011) | 0.001  (0.011) | 0.025^**^  (0.011) | 0.034  (0.055) | 0.044  (0.056) | 0.039  (0.052) | 0.059  (0.090) | 0.075  (0.091) | 0.069  (0.082) |
| *ln(Livestream_it_)* | 0.007  (0.010) | 0.009  (0.010) | 0.005  (0.019) | 2.110^***^  (0.053) | 2.109^***^  (0.053) | 0.641^**^  (0.091) | 3.452^***^  (0.086) | 3.451^***^  (0.086) | 0.566  (0.143) |
| *Popularity* | - | 0.561^***^  (0.041) | - | - | -0.236  (0.216) | - | - | -0.391  (0.348) | - |
| *lnPV* | - | - | 0.000  (0.003) | - | - | 0.279^***^  (0.014) | - | - | 0.548^***^  (0.023) |
| Creator FE | Yes | Yes | Yes | Yes | Yes | Yes | Yes | Yes | Yes |
| Time FE | No | No | No | No | No | No | No | No | No |
| Number of Users | 123 | 123 | 123 | 123 | 123 | 123 | 123 | 123 | 123 |
| Number of Periods | 25 | 25 | 25 | 25 | 25 | 25 | 25 | 25 | 25 |
| Observations | 3075 | 3075 | 3075 | 3075 | 3075 | 3075 | 3075 | 3075 | 3075 |
| Adjusted *R*^2^ | 0.016 | 0.076 | 0.016 | 0.388 | 0.388 | 0.457 | 0.389 | 0.389 | 0.491 |

Notes: standard errors are in parentheses. * *p* < 0.1; ** *p* < 0.05; *** *p* < 0.01.

**Table 6** The Impact of Content Coverage (Common Time Trend Uncontrolled)

| **Explanatory Variable** | **Explained Variable** | | |
| --- | --- | --- | --- |
|  | ln(*Follow_it_*) | ln(*Order_it_*) | ln(*TR_it_*) |
| *ContentCoverage* | 0.018^**^  (0.007) | 0.013  (0.017) | 0.016  (0.029) |
| Creator FE | Yes | Yes | Yes |
| Time FE | No | No | No |
| Number of Users | 123 | 123 | 123 |
| Number of Periods | 6 | 6 | 6 |
| Observations | 738 | 738 | 738 |
| Adjusted *R*^2^ | 0.071 | 0.023 | 0.019 |
| Notes: standard errors are in parentheses. * *p* < 0.1; ** *p* < 0.05; *** *p* < 0.01. | | | |

**Table 7** The Impact of Content Type (Common Time Trend Uncontrolled)

| **Explanatory Variable** | **Explained Variable** | | |
| --- | --- | --- | --- |
|  | ln(*Follow_it_*) | ln(*Order_it_*) | ln(*TR_it_*) |
| *ln(SciContent_it_)* | 0.122^***^  (0.036) | 0.265  (0.170) | 0.221  (0.291) |
| *ln(DietContent_it_)* | 0.178^***^  (0.021) | -0.070  (0.098) | -0.113  (0.167) |
| *ln(AdvContent_it_)* | -0.02  (0.006) | 0.140^***^  (0.028) | 0.152^***^  (0.047) |
| Creator FE | Yes | Yes | Yes |
| Time FE | No | No | No |
| Number of Users | 123 | 123 | 123 |
| Number of Periods | 6 | 6 | 6 |
| Observations | 738 | 738 | 738 |
| Adjusted *R*^2^ | 0.225 | 0.087 | 0.072 |
| Notes: standard errors are in parentheses. * *p* < 0.1; ** *p* < 0.05; *** *p* < 0.01. | | | |

**Table 8** Moderating Effect of Creators’ Characteristics (Common Time Trend Uncontrolled)

| **Explanatory Variable** | **Model 1** | | | **Model 2** | | |
| --- | --- | --- | --- | --- | --- | --- |
|  | ln(*Follow_it_*) | ln(*Order_it_*) | ln(*TR_it_*) | ln(*Follow_it_*) | ln(*Order_it_*) | ln(*TR_it_*) |
| *ContentCoverage_it_* | 0.018^**^  (0.003) | 0.013  (0.018) | 0.016  (0.030) | -0.002  (0.005) | - | - |
| *Occupation_i_* | - | - | - | - | - | - |
| *ContentCoverage_it_* Occupation_i_* |  |  |  | 0.030^***^  (0.006) | - | - |
| Creator FE | Yes | Yes | Yes | Yes | - | - |
| Time FE | Yes | Yes | Yes | Yes | - | - |
| Observations | 738 | 738 | 738 | 738 | - | - |
| Adjusted *R*^2^ | 0.071 | 0.023 | 0.019 | 0.107 | - | - |
| **Explanatory Variable** | **Model 3** | | | **Model 4** | | |
|  | ln(*Follow_it_*) | ln(*Order_it_*) | ln(*TR_it_*) | ln(*Follow_it_*) | ln(*Order_it_*) | ln(*TR_it_*) |
| *ln(SciContent_it_)* | 0.122^***^  (0.036) | 0.265  (0.170) | 0.221  (0.291) | 0.018  (0.030) | 0.343^*^  (0.196) | 0.472  (0.321) |
| *ln(DietContent_it_)* | 0.178^***^  (0.021) | -0.070  (0.098) | -0.113  (0.167) | 0.058^***^  (0.034) | -0.196  (0.164) | -0.287  (0.280) |
| *ln(AdvContent_it_)* | -0.02  (0.006) | 0.140^***^  (0.028) | 0.152^***^  (0.047) | -0.016  (0.039) | 0.611^***^  (0.190 | 0.773^**^  (0.324) |
| *Occupation_i_* | - | - | - | - | - | - |
| *ln(SciContent_it_)*Occupation_i_* | - | - | - | 0.139^***^  (0.050) | - | - |
| *ln(DietContent_it_)*Occupation_i_* | - | - | - | 0.173^***^  (0.027) | - | - |
| *ln(AdvContent_it_)*Occupation_i_* | - | - | - | - | 0.335  (0.230) | 0.817^**^  (0.376) |
| Creator FE | Yes | Yes | Yes | Yes | Yes | Yes |
| Time FE | Yes | Yes | Yes | Yes | Yes | Yes |
| Observations | 738 | 738 | 738 | 738 | 738 | 738 |
| Adjusted *R*^2^ | 0.225 | 0.087 | 0.072 | 0.221 | 0.049 | 0.046 |
| **Explanatory Variable** | **Model 5** | | | **Model 6** | | |
|  | ln(*Follow_it_*) | ln(*Order_it_*) | ln(*TR_it_*) | ln(*Follow_it_*) | ln(*Order_it_*) | ln(*TR_it_*) |
| *ContentCoverage_it_* | 0.018^**^  (0.003) | 0.013  (0.018) | 0.016  (0.030) | 0.025^***^  (0.003) | - | - |
| *Type1_i_* | - | - | - | - | - | - |
| *Type2_i_* | - | - | - | - | - | - |
| *ContentCoverage_it_* Type1_i_* | - | - | - | -0.004  (0.036) | - | - |
| *ContentCoverage_it_* Type2_i_* | - | - | - | -0.033^***^  (0.007) | - | - |
| Creator FE | Yes | Yes | Yes | Yes | - | - |
| Time FE | Yes | Yes | Yes | Yes | - | - |
| Observations | 738 | 738 | 738 | 738 | - | - |
| Adjusted *R*^2^ | 0.071 | 0.023 | 0.019 | 0.101 | - | - |
| **Explanatory Variable** | **Model 7** | | | **Model 8** | | |
|  | ln(*Follow_it_*) | ln(*Order_it_*) | ln(*TR_it_*) | ln(*Follow_it_*) | ln(*Order_it_*) | ln(*TR_it_*) |
| *ln(SciContent_it_)* | 0.122^***^  (0.036) | 0.265  (0.170) | 0.221  (0.291) | 0.148^***^  (0.047) | -0.251  (0.221) | -0.625^*^  (0.361) |
| *ln(DietContent_it_)* | 0.178^***^  (0.021) | -0.070  (0.098) | -0.113  (0.167) | 0.058^***^  (0.017) | -0.028^**^  (0.014) | -0.159  (0.180) |
| *ln(AdvContent_it_)* | -0.02  (0.006) | 0.140^***^  (0.028) | 0.152^***^  (0.047) | -0.024  (0.018) | 0.115^**^  (0.053) | 0.649^***^  (0.249) |
| *Type1_i_* | - | - | - | - | - | - |
| *Type2_i_* | - | - | - | - | - | - |
| *ln(SciContent_it_)* Type1_i_* | - | - |  | -0.153^***^  (0.054) | - | - |
| *ln(SciContent_it_)* Type2_i_* | - | - |  | -0.153^***^  (0.055) | - | - |
| *ln(DietContent_it_)* Type1_i_* | - | - |  | -0.115^**^  (0.049) | - | - |
| *ln(DietContent_it_)* Type2_i_* | - | - |  | -0.116^**^  (0.050) | - | - |
| *ln(AdvContent_it_)* Type1_i_* | - | - | - | - | 0.145  (0.661) | -0.257  (1.081) |
| *ln(AdvContent_it_)*Type2_i_* | - | - | - | - | -0.086  (0.218) | -0.333  (0.357) |
| Creator FE | Yes | Yes | Yes | Yes | Yes | Yes |
| Time FE | Yes | Yes | Yes | Yes | Yes | Yes |
| Observations | 738 | 738 | 738 | 738 | 738 | 738 |
| Adjusted *R*^2^ | 0.225 | 0.087 | 0.072 | 0.173 | 0.055 | 0.051 |

Notes: standard errors are in parentheses. * *p* < 0.1; ** *p* < 0.05; *** *p* < 0.01.
